# Progress in Diseases of the Nervous System

**Published:** 1903-05-02

**Authors:** 


### Progress in Diseases of the Nervous System.

**Suture of the Brachial Plexus in Birth Paralysis of the Upper Extremity.**—Kennedy [1] describes operation in three cases of birth palsy. In this affection, as first described by Duchenne, the arm at birth hangs powerless by the side and cannot be abducted, the forearm is extended and cannot be flexed, and there is very marked pronation, so that the hand looks away from the side. This position of the limb is caused by paralysis of the following muscles: the deltoid and supraspinatus, the biceps, brachialis anticus and supinator longus, the supinator brevis and infraspinatus and teres minor. Erb described a paralysis occurring in adults with the same characters as Duchenne, and his investigations enabled him to locate the exact spot which, when stimulated, would lead to a contraction of the affected muscles, and this spot corresponded to the position of the anterior primary divisions of the fifth and sixth cervical nerves where they unite to contribute to the brachial plexus. In some way, therefore, during the birth of the child the upper part of the brachial plexus must be compressed, stretched, or torn. As a rule there is the history of considerable force having been applied during the birth of the child, and whatever the presentation may have been the chief factor probably is forcible depression of the shoulder while the head is bent to the opposite side and rotated. It is difficult to say whether there is any loss of sensation owing to the difficulty in testing young infants. The usual treatment for these cases is massage, electricity, and passive movements. Some of the cases quite recover, some improve, and others show no sign of improvement. The writer considers this treatment of little value as some cases recover with no treatment at all. He holds the only rational thing is to treat them in the same way as injuries to the peripheral nerves in general. As the injury may be only slight and may be recovered from, a sufficient length of time must elapse before operation to see if the developing electrical reactions indicate an approaching recovery of the muscles. During the first few weeks of life the electrical reactions are unsatisfactory, but by two months good contractions can be obtained by the Faradic current. If a month or two after this contractions can still be obtained, the case will probably recover, but if not, the operation should be performed. The following are the chief steps: an incision is made across the posterior triangle from the middle of the sterno-mastoid to the outer part of the clavicle, the superficial fascia, platysma, and deep fascia are divided, the omo-hyoid exposed and the nerves dissected out above this muscle. The junction of the fifth and sixth is found by tracing the upper trunk inwards. The branches are cleared, the supra-scapular and the branches to the outer and posterior cords of the plexus freed from adhesions, the cicatricial portion excised until healthy nerve fibres are seen in both ends and the divided ends of the fifth and sixth cervical nerves sutured with catgut to the cut surfaces of supra-scapular and branches to outer and middle cords. The shoulder is approximated to the neck during the suturing and kept fixed there for a fortnight during the healing of the wound. The writer's first case was a child a little over two months old; no improvement was observed for three months. Nine months after the operation nearly all the movements except supination had returned. The second case was fourteen years old and there was marked muscular atrophy. Five months after operation there was no improvement in movement but Faradic contractions were improved. The third case was operated on at six months old, but time had not elapsed for any signs of recovery to be present.

**Theory and Practice of Spinal Cocainisation.**—Dickinson [2] contributes this paper. In a brief historical review he shows that Corning in 1885, from a series of laboratory experiments, was the first to suggest the possibility of its replacing etherisation. His observations were, however, unnoticed until 1899, when Bier and Juffier put it into actual practice. The writer then describes the anatomy of the region punctured by the needle to reach the spinal membranes, and he points out that the condensed fibrous tissue of the deeper parts may be so firm as to give one the impression that the bone has been reached before this is actually the case. The arrangement of the cord and nerves in the spinal canal follows, and as a result of experiment it would appear that there is usually a quarter of an inch of fluid separating the ligamentum subflavum from the nerves composing the cauda equina. If these are pricked there is a sensation of pain or distress in one or other foot. To perform the little operation the skin must be thoroughly cleansed and frozen and a small puncture made with a bistoury at a point a quarter of an inch below and half an inch to the right of the third lumbar spine the needle is entered through the wound pointing to "five o'clock" directing the point a little upwards and towards the ligamentum subflavum. Fluid will flow, and about twice as much should be allowed to escape as is going to be thrown in. Half a grain is the quantity of cocaine to be injected and it is of the greatest importance that this should be perfectly pure. The local effect begins at once and in from three to five minutes there is complete analgesia of the superficial nerves below the puncture. In five minutes more the deeper nerves are affected in the same way. The strength of the solution diminishes as it spreads through the space, so that the analgesia becomes surgically more uncertain as it spreads upwards. At the mammary line it generally stops for surgical purposes. In 20 minutes systemic results may be expected. The skin blanches, the cerebrum becomes anæmic, and vomiting results—involuntary defecation may follow. The analgesia appears first in the pudendal region or in the feet. Areas of analgesia coalesce until the complete effect is produced. If the cocaine pass to the medulla cardio-respiratory effects of panting and rapid heart action with a sense of suffocation may be observed. The rectal temperature may be raised from one to five degrees. Some complain of headache. A severe ache in the back and thigh is a more constant effect, and may persist several hours after the analgesia has passed off. The writer goes on to say that the cardio-respiratory phenomena are those mainly to be apprehended, but questions whether they can follow a half-grain dose. In an experience of over 200 cases there was not once any distress from dyspnœa or palpitation. Blanching of the skin and nausea were observed in a few cases, but less in the later ones, when more cerebro-spinal fluid was removed before the injection. The cocaine should be sterilised by submitting it to a tmperature of 176° for four hours for four successive days; a temperature of 180° will destroy it. The most concern must be felt from idiosyncrasy. Cocaine anæsthesia is to be preferred to ether in all cases where the patient will not be over-much mentally disturbed by the surroundings. Many more cases, however, must be carefully observed before it can be rated with ether or chloroform in safety. It is not proven to carry enough risk to prohibit careful clinical experimentation. Surgical analgesia of the lower limbs will be attained in three to five minutes in the great majority of cases, but for operations on the mamma or head thirty minutes must elapse.

**A Treatment for Spina Bifida.**—Griffith [3] quotes a case in which he operated. At the time of birth a soft hollow spot was noticed in the small of the back covered with white skin. Directly after birth a tumour formation rapidly took place over the lower third of the back. At three weeks old the infant came under the care of the writer. The tumour was then fifteen inches in circumference and three inches high; the covering was like stretched parchment and ulcerated on the top. This ulceration healed under treatment with boracic ointment; and when the child was three months old the first sign of leaking appeared. Operation was at once undertaken. The child was placed in a position with its head downwards, the fluid was drawn off with a trocar, and an incision made through the tumour on one side and parallel to the spine, completely opening the sac. At the bottom of the sac was a circular orifice filled up with membranous tissue through which appeared the stump of the spinal cord. The sides of the wall were trimmed away, no attempt at any osteoplastic work being made owing to the child's condition. These side walls were sutured together over sterile gauze, which was removed before the last stitches were tied. A dressing of gauze was fixed on by plaster passing two-thirds round the body. A good deal of cerebro-spinal fluid escaped for the first day or two, but the child appeared to be doing well up to the fifth day, when the mother let the child fall, with the result that it became collapsed, and died in a few hours.

**Retinal Hæmorrhages as a Diagnostic Feature in Fracture of the Base of the Skull.**—Dr. Fleming,[4] after describing the vascular supply of the optic nerve and retina and showing the close communication between the perivascular lymph spaces and the subarachnoid space, mentioned 12 cases of fracture of the skull, all except one being of the base, in which, where the hæmorrhage into the subarachnoid space was acute, retinal hæmorrhages were almost invariably present, and further that where the subarachnoid hæmorrhage was unilateral the retinal hæmorrhages were mostly confined to the affected side. All the cases which showed no retinal hæmorrhages were less acute. Further, in four cases of intra-cerebral hæmorrhage in which there was no fracture there were retinal hæmorrhages in three associated with very sudden and considerable subarachnoid hæmorrhage. All these were unilateral, and the effusion into the subarachnoid space was mainly on one side. All these observations were made in the post-mortem room.

**Cerebro-spinal Rhinorrhœa after Fracture of the Skull.**[5]—Two cases of this affection are quoted from Larkin. In the first a man fell 15 feet on to his face, lost consciousness, and vomited; was admitted to hospital with concussion, regained consciousness in two days, and complained of deafness; had purulent otitis, which lasted a week. At three weeks from accident complained that handkerchief was saturated with fluid from nose, and on stooping clear watery fluid flowed from nose, chiefly left side. Unconsciousness and symptoms of meningitis followed, and death occurred on the twenty-seventh day from the accident. The necropsy showed a slit in the basi-sphenoid three-quarters of an inch long, through which a probe could be passed into the nose. In another case, as a result of a bicycle accident, there was a fracture of the frontal bone extending into the fronto-ethmoid region. Recovery took place from the injury and the patient resumed work, but was annoyed by a watery discharge from the nose. This stopped suddenly in six weeks, and patient remained well except for occasional headache. Nine months after he died of meningitis. In a third case, reported by Bosworth, there was fracture of the cribriform plate, and death occurred on the eighteenth day after the injury.

**Tumours of the Optic Nerve.**—Parsons[6] read a paper on primary extradural tumours of the nerve. They are much less frequent than the intradural, usually commence before the age of 10; the prominent symptom is exophthalmos, the failure of vision slow and accompanied by optic neuritis of the "choked disc" variety. In no case was the globe invaded by the growth. Eight out of 18 growths were endotheliomata, several having characteristics of psammomata. They were of slow growth and low malignancy, and wherever possible Krönlein's operation with retention of the globe was indicated. Werner[7] reported two cases of tumour of the optic nerve removed by Krönlein's method. The first, a woman of 45, had noticed a swelling of the inner canthus for a year. When seen the left eye was two centimetres in advance of the other, no pulsation or bruit. Eye blind with marked optic atrophy, and anæsthetic. A semi-circular incision convex forwards, extending to near outer canthus was made to expose outer wall of orbit. The bone was divided at fronto-malar suture and just above zygoma extending incision backwards to spheno-maxillary fissure, and the bony mass turned back with the soft parts. The tumour was isolated and removed. Posteriorly it involved the apex of the orbit, and had to be removed piecemeal, but in front there was a clear portion of optic nerve. The tumour inclosed in the dural sheath was an alveolar sarcoma; the arrangement of the cells suggested endothelioma. The second case was a girl of 11 years whose eye had been prominent and divergent for 14 months. The eye was removed with the growth and the orbit cleared out. The growth was a myxo-sarcoma surrounding the nerve. Krönlein first performed this operation in 1886 and published an account in 1889. The risk is small, there having been one death in 73 cases.

**Surgical Intervention in Cerebral Hæmorrhage.**—Lambotte[8] reports two cases of cerebral hæmorrhage treated by trephining. In the first a hæmorrhagic cavity was exposed in the right parietal lobe and several clots and detritus of cerebral substance removed, rapid and complete cure resulting. In the second case no clot could be found but benefit followed from relief of intra-cranial pressure. The author does not advise ligature of the common carotid before trephining. The cranium should be trephined over the fissure of Sylvius, the dura mater incised and the brain punctured by an exploring needle in the direction of the internal capsule. If a clot be found it should be freely exposed by incision of the cerebral substance and the cavity drained by gauze.

[1] Brit. Med. Jour., Feb. 7. [2] Med. Rec., Feb. 7. [3] Med. Rec., Jan. 31. [4] Brit. Med. Jour.. Feb. 21. [5] Lancet, Feb. 14. [6] Ibid. [7] Ibid. [8] Brit. Med. Jour., Epit., Dec. 6, 1902.

